# Efficacy of Botulinum Toxin Type A for Prevention of Post-Mastectomy Scar in Transmen: A Prospective, Randomized Study

**DOI:** 10.3390/toxins15110636

**Published:** 2023-10-31

**Authors:** Waranaree Winayanuwattikun, Vasanop Vachiramon, Teerapong Rattananukrom, Pasita Palakornkitti, Ngamcherd Sitpahul

**Affiliations:** 1 Division of Dermatology, Faculty of Medicine Ramathibodi Hospital, Mahidol University, Bangkok 10400, Thailand; waranaree.win@mahidol.ac.th (W.W.); teerapong.rat@mahidol.ac.th (T.R.); pasita.pal@mahidol.ac.th (P.P.); 2 Department of Plastic and Maxillofacial Surgery, Faculty of Medicine Ramathibodi Hospital, Mahidol University, Bangkok 10400, Thailand; ngamcherd.sit@mahidol.edu

**Keywords:** injectables, neuromodulators, neurotoxin, surgical scars

## Abstract

Background: Subcutaneous mastectomies in transmen have been gaining popularity. However, post-operative scars are an inevitable consequence. Recently, Botulinum neurotoxin A (BoNT-A) has shown positive effects in scar prevention. The objective of this study is to investigate the effectiveness of BoNT-A in scar prevention. Methods: Fifteen patients who had undergone subcutaneous mastectomy were included. At 14 days post-surgery, either incoBoNT-A or a placebo was injected into the scar on each side. The primary outcome assessment measured the scar’s severity using the Vancouver Scar Scale (VSS) and the Patient and Observer Scar Assessment Scale (POSAS). The secondary outcome assessment evaluated the scar’s color using a standard measurement device. Outcome assessments were conducted until 6 months post-surgery. Results: There were significantly lower VSS scores in the BoNT-A group compared to the placebo at the end of the study (7.43 ± 0.26 vs. 8.82 ± 0.26, *p* < 0.001). The objective assessment revealed a statistically significant decrease in redness values in the BoNT-A group compared to the placebo at 3 and 6 months. Conclusion: BoNT-A has demonstrated effectiveness in scar prevention by reducing the severity of postoperative scar formation and improving overall scar appearance.

## 1. Introduction

Gender dysphoria is defined as the conflict between biological sex and gender identity [1]. Individuals with this condition often experience distress and require medical care [1,2]. Many studies address the health issues that relate to gender dysphoria such as HIV and other sexually transmitted infections, mental health conditions such as depression and anxiety, as well as substance abuse [1,2]. Various treatment options are available, including psychological support, hormonal substitution, and sexual reassignment surgery [3]. Management of this condition should involve a combined and multidisciplinary approach by healthcare professionals. A survey in the United States indicated a rising trend in gender affirmation surgeries over time [4,5]. The increase in transgender surgeries is part of a broader movement towards recognizing and respecting the rights and identities of transgender people. Among transmen, dissatisfaction often centers around their breasts [6], leading to an increased demand for chest reconstruction surgery or bilateral mastectomy to achieve a more masculine appearance [4,7]. However, post-surgical scarring is a common outcome. Mastectomy procedures have been documented to address post-surgical scars, which often necessitating scar revision [8,9]. In a retrospective study of female-to-male subcutaneous mastectomies, 12.6% required scar revision [8].

Scarring arises from the excessive proliferation of fibroblasts during wound healing [10]. Beyond the undesirable cosmetic impact, scars can induce symptoms like pain, itching, and general discomfort [11]. Various approaches, including steroid injections, laser therapy, compression techniques, and surgical interventions, have been employed to manage these concerns. However, the effectiveness of these treatments remain suboptimal, and certain methods might carry potential side effects for patients [10,12].

Botulinum toxin, produced by *Clostridium botulinum*, is commonly recognized as a neurotoxin. Its ability to hinder the release of the neurotransmitter acetylcholine from axons at the neuromuscular junction, thereby temporarily impeding muscle contractions, has long been understood. Presently, there are seven serotypes (A, B, C1, D, E, F, G). While serotypes A and B are employed in clinical applications, botulinum neurotoxin A (BoNT-A) is the most frequently used variant.

Zimbler et al. proposed the effectiveness of BoNT-A in impeding scar formation at surgical sites through the concept of wound immobilization [13]. By inducing muscle paralysis, the wound environment is devoid of the adverse influence of muscle tension on the healing process [13,14]. This concept was supported by Chen et al., who conducted a systematic review investigating the relationship between BoNT-A and post-surgical scars, affirming its efficacy and safety in scar prevention [15]. In previous studies, the efficacy of onaBoNT-A or aboBoNT-A for the prevention of post-surgical scars has been demonstrated [13,14,15]. However, the data regarding the efficacy of incoBoNT-A for the scar prevention are limited.

This randomized controlled study is designed to assess the efficacy and safety of incoBoNT-A for the prevention of abnormal scar formation following subcutaneous mastectomy in transmen.

## 2. Results

**Figure 1:**
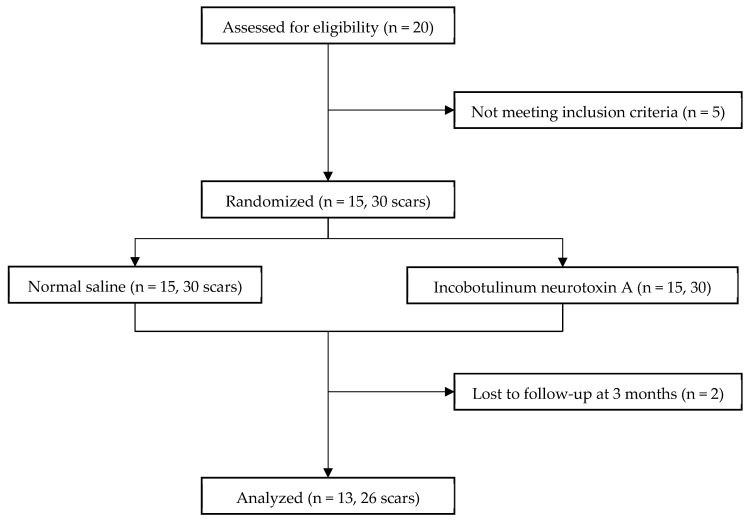
Consolidated standard of randomized trials (CONSORT) diagram of this study.

**Figure 2:**
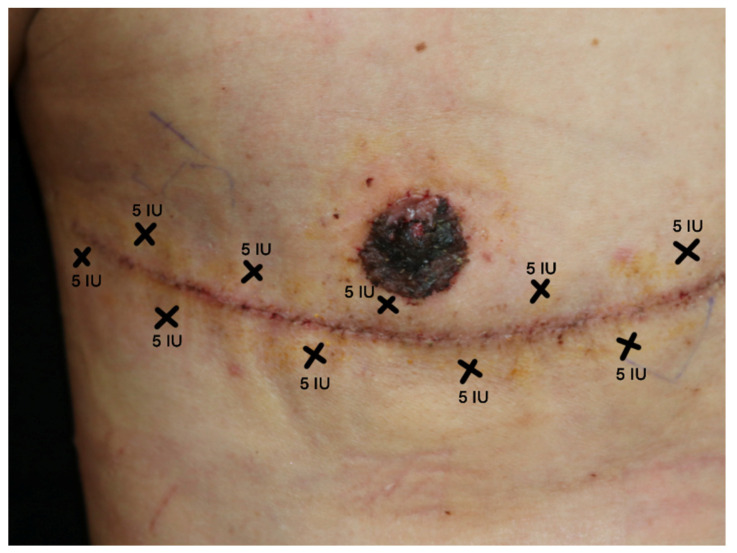
Pattern of incoBoNT-A injection to post-surgical scar. This figure demonstrates the pattern of injection for botulinum neurotoxin A. The crisscross marks reflect the injection sites which were 1 cm away from the surgical scar. Each point of injection was 5 units for a total of 50 units.

The study included 15 female-to-male patients. Thirteen patients (accounting for 26 surgical scars) successfully completed the study until its conclusion, while 2 patients were lost to follow-up at the 3-month mark because of personal inconvenience with the follow-up schedule; the attrition did not relate to the treatment protocol or any adverse events (Figure 1). Pattern of incoBoNT-A injection is shown in Figure 2 (more details are discussed in materials and methods section). Baseline patient characteristics are presented in Table 1.

Notably, all patients were right-hand dominant. Hand-side dominance may indirectly contribute to an increased tendency for scar formation on the dominant side. This is because the dominant side often experiences greater skin tension, muscle contraction, and movement. Among the participants, one patient had a history of premature ventricular contractions and was currently taking medication. Additionally, all patients had Fitzpatrick phototype 3 or 4. A single patient had a previous history of keloid formation on their right hand. The baseline assessment scores between the two groups, as shown in Table 2, demonstrated no significant differences.

### 2.1. VSS Results of BoNT-A Treatment

**Figure 3:**
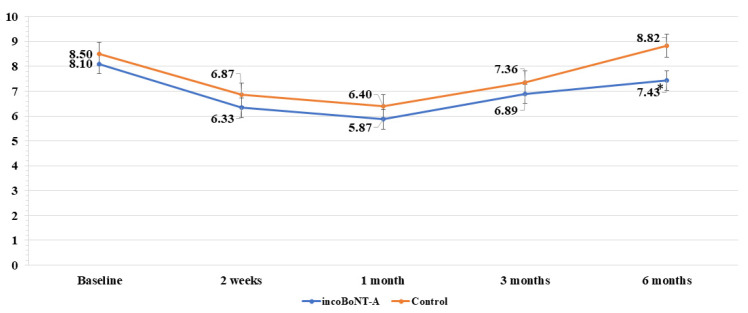
VSS scores for the incoBoNT-A and control group. Abbreviations: incoBoNT-A, Incobotulinum neurotoxin A; VSS, Vancouver scar scale; * *p* value < 0.05.

The VSS scores in both groups exhibited a gradual reduction up to the 1-month mark, as shown in Figure 3. Subsequently, after the 3-month point, scores increased in both groups. Nevertheless, at the conclusion of the study, significantly lower scores were observed in the incoBoNT-A group in comparison to the control group (7.43 ± 0.26 vs. 8.82 ± 0.26, *p* < 0.001).

### 2.2. POSAS Results of incoBoNT-A Treatment

**Figure 4:**
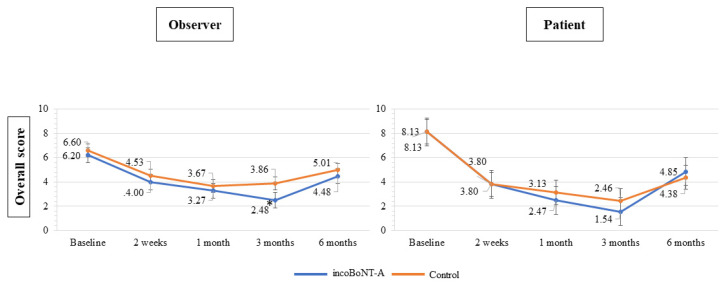
POSAS scores for the incoBoNT-A and control group. Abbreviations: incoBoNT-A, Incobotulinum neurotoxin A; POSAS, patient and observer scar assessment scale; * *p* value < 0.05.

Likewise, the POSAS scores in both the observers’ and patients’ sections demonstrated an increase after the 3-month mark (Figure 4). The overall observer scores in the incoBoNT-A group were significantly lower than those in the control group at 3 months (2.48 ± 0.23 vs. 3.86 ± 0.23, *p* < 0.001). Regarding the patients’ rating scale section, no significant differences in scores between the two groups were observed over the course of the entire study period.

### 2.3. Scar Color Assessment by Colorimeter

**Figure 5:**
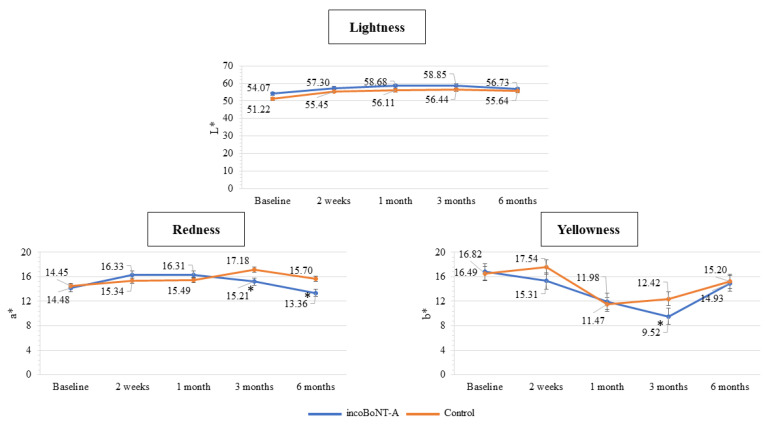
Scar color assessment by Colorimeter for the incoBoNT-A and control group. Abbreviations: incoBoNT-A, Incobotulinum neurotoxin A; * *p* value < 0.05.

The Colorimeter was used to assess the scars’ lightness, redness, and yellowness, as illustrated in Figure 5.

A statistically significant decrease in the redness value of the incoBoNT-A group was observed compared to the control group at 3 and 6 months (15.21 ± 0.50 vs. 17.18 ± 0.50, *p* = 0.005 and 13.36 ± 0.50 vs. 15.70 ± 0.50, *p* = 0.001, respectively). Furthermore, the incoBoNT-A group exhibited statistically lower yellowness values in comparison to the control group at 3 months (9.52 ± 0.06 vs. 12.42 ± 0.96, *p* = 0.032). Although the treated sides showed higher lightness values than those receiving the placebo, no significant statistical difference was observed.

**Figure 6:**
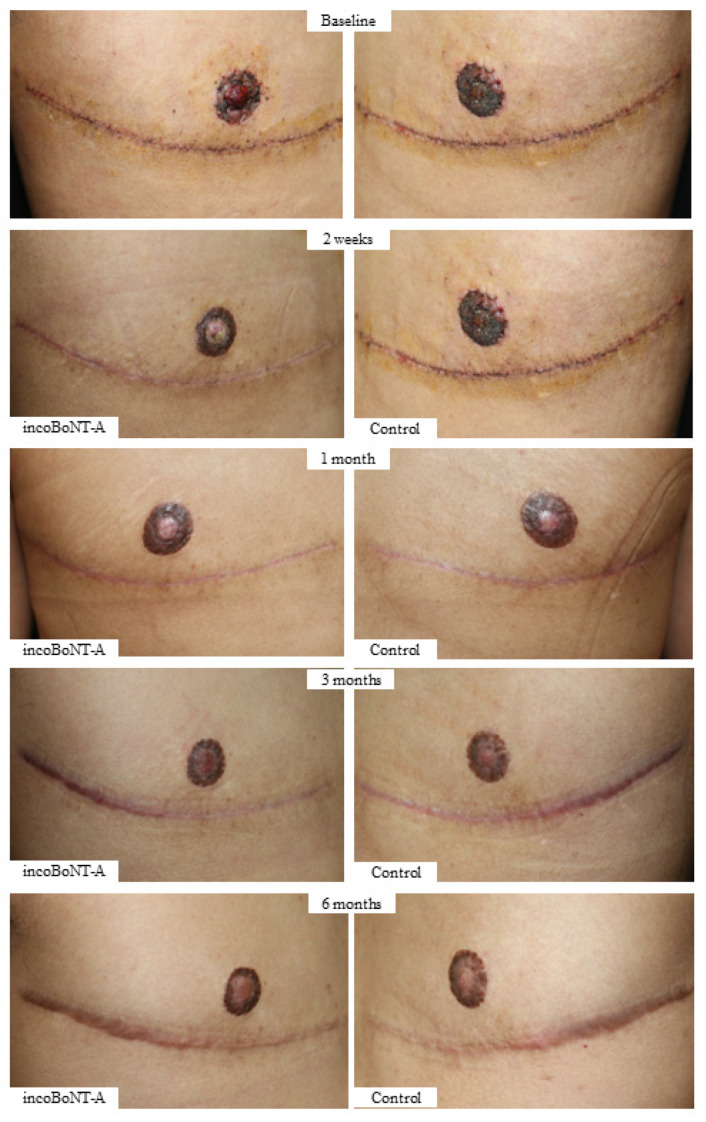
Representative case. Representative before-and-after photographs of a 28-year-old female-to-male patient. Fifty units of incoBoNT-A were administered on the right surgical scar. Abbreviations: incoBoNT-A, Incobotulinum neurotoxin A.

At the end of the study, patients in the incoBoNT-A group reported significantly higher satisfaction scores compared to the control group (8.88 ± 0.36 vs. 8.74 ± 0.52, *p* = 0.001). Throughout the study duration, no adverse events were reported. Figure 6 illustrates a representative case from the study. For detailed comparisons between the two groups, please refer to Table 3, which presents all the measurement data.

## 3. Discussion

Health issues associated with chest binding have been documented among individuals [16]. Transmen commonly express greater satisfaction with their appearance after undergoing mastectomy surgery to achieve a more masculine chest [6]. However, there have been reports of secondary scar revision procedures [9]. Achieving desirable aesthetic outcomes in the chest area following surgery can be complex, primarily because of the region’s high-tension nature that makes it susceptible to scar formation [17]. Unfavorable scarring in this region has been identified in approximately 38% to 68% of cases postoperatively [17], with around 60% of such cases occurring within 3 months after surgery [18].

Recently, Botulinum toxin type A (BoNT-A) has shown promising effectiveness in scar prevention, and its underlying mechanisms have been extensively explored. The scar prevention benefits of BoNT-A have initially been attributed to the immobilization of the wound through temporary paralysis of underlying muscles [19]. Subsequently, additional non-neuronal mechanisms contributing to scar prevention have been identified through both in vitro and in vivo studies.

To begin with, BoNT-A is known to down-regulate the expression of transforming growth factor-beta 1 (TGF-β1), leading to reduced fibroblast proliferation and differentiation [20]. This effect has been demonstrated by Kim et al., who observed decreased collagen accumulation in scars treated with BoNT-A [21]. Furthermore, BoNT-A exerts an anti-inflammatory impact by diminishing inflammatory cells and cytokine expression during the inflammatory phase [20,22]. Lastly, research conducted on animal models has unveiled BoNT-A’s effects on the vascular endothelium. It induces vasodilation, thereby increasing blood flow to vessels, and enhances the production of vascular growth factors [23]. These multifaceted effects collectively explain how BoNT-A contributes to tissue healing and scar prevention.

To the best of our knowledge, this is the first randomized, double-blind, placebo-controlled study employing incoBoNT-A administration for scar prevention following female-to-male subcutaneous mastectomy. In our study, a separate-scar side design was employed, wherein a single patient served as both an intervention and a control group, effectively mitigating confounding factors in scar formation.

Employing subjective scar assessments, the Vancouver Scar Scale (VSS) and the Patient and Observer Scar Assessment Scale (POSAS), a trend of increasing scores was observed at the 3-month mark. Because of natural wound healing and remodeling processes, around 3 months post-surgery there is an increase in collagen production, which is a key component of scar tissue. This trend mirrors the incidence rate of hypertrophic scars, which was approximately 60% after procedures like mammoplasty or median sternotomy at 3 months [18].

Regarding the subjective evaluations using VSS and POSAS, our study’s outcomes indicated that only the VSS score at the study’s conclusion was significantly lower in the incoBoNT-A group in comparison to the control group. Above all, patients reported significantly higher satisfaction with the incoBoNT-A injected side compared to the control side at the end of the study.

According to a recent systematic review by Zhang et al., 12 studies were included that investigated the postoperative administration of BoNT-A [24]. The results from meta-analysis demonstrated that BoNT-A exhibited positive effects in enhancing postoperative surgical scars, as assessed by various methods including the Visual Analog Scale (VAS) score, Vancouver Scar Scale (VSS) score, and patient satisfaction. However, the Patient and Observer Scar Assessment Scale (POSAS) yielded inconsistent findings across different studies [19,21,25]. Our study’s findings align with this meta-analysis, as we observed improvements in VSS scores and higher patient satisfaction in the BoNT-A group, while no significant difference was detected in the POSAS score.

Li et al. conducted a split-scar study on the chest wall area and reported significant improvements in the VSS score and patient satisfaction in the BoNT-A group compared to the control group at the 6-month follow-up [26]. Similarly, a recent study by Abedini et al. focused on mammoplasty and abdominoplasty scars, employing a modified Stony Brook Scar Evaluation Scale (SBSES), and identified better scores in the BoNT-A group at both 3 and 6 months [27].

For a deeper comprehension of the non-neural mechanisms associated with the use of BoNT-A in scar management, it’s crucial to examine its impact on the wound healing process. One well-documented effect of BoNT-A is its ability to down-regulate the expression of Transforming Growth Factor-Beta 1 (TGF- β1), which in turn reduces fibroblast proliferation and differentiation [20]. This has been demonstrated in a study by Kim et al., where scar tissue injected with BoNT-A exhibited reduced collagen accumulation [21]. Additionally, BoNT-A exerts an anti-inflammatory effect by decreasing inflammatory cell presence and cytokine expression during the inflammatory phase [20,22]. Lastly, animal studies have explored the effects of BoNT-A on vascular endothelium. It is found to increase blood flow through vasodilation and enhance the production of vascular growth factors [23]. These mechanisms collectively highlight the beneficial role of BoNT-A in tissue healing and scar prevention.

Several studies have examined the effect of BoNT-A on gene expression, and these studies have found that BoNT-A can inhibit particular genes that relate to scar formation. One important gene is PTEN (Phosphatase and Tensin homolog), which plays a crucial role in glial scar formation [28]. BoNT-A has been shown to stimulate PTEN negative regulation. After PTEN was activated, it sent signals that reduce the activity of the PI3K and Akt genes, ultimately stopping the growth of fibroblast cells [29]. Additionally, BoNT-A can decrease the production of the cyclin D1 gene, which, in turn, promotes the apoptosis of fibroblast cells [30]. Another gene called Growth Arrest and DNA Damage-Inducible gene 153 (GADD153) play a big role in a process where cells in the endoplasmic reticulum can cause other cells to die. In a study by Nien [31], they showed that a substance called BoNT-A can make the GADD153 protein become more active in fibroblast cells found in keloid scars. This happens through a pathway called JNK/MAPK, and it leads to the death of fibroblast cells.

In terms of objective evaluation, the redness of scars assessed by Colorimeter displayed statistically lower values in the incoBoNT-A group compared to the control group at 3 and 6 months. This finding could indicate the efficacy of incoBoNT-A in reducing inflammation and assisting immature scars in maturing with a diminished degree of discoloration [32]. Lee et al. conducted quantitative measurements of color differences and concluded that the degree of discoloration in the BoNT-A group was superior to that in the control group at the 6-month mark [33].

Overall, our study underscores that incoBoNT-A has the potential to ameliorate both scar severity and appearance. Over the entire study duration, no serious adverse events were observed. Furthermore, while patients did not regain normal sensation in the surgical scar area because of the operation, local pain during injection was manageable.

It is important to acknowledge that scar formation can be influenced by various factors, including different anatomical locations and ethnicities. Notably, many of the positive studies on scar improvement have focused on facial scars [34,35]. In contrast, our study specifically targeted surgical scars located on the chest, which is recognized as a high-risk region for scar formation. The anterior chest area experiences heightened skin tension due to repetitive horizontal stretching caused by upper limb movements. Moreover, movements involving body bending can also influence skin tension in this region [17]. Additionally, our study was conducted within an Asian patient population who are known to be more susceptible to developing keloids and hypertrophic scars [34,36]. Despite these challenges, our study’s findings demonstrated the favorable effects of incoBoNT-A in preventing undesirable postoperative surgical scars.

This study does have certain limitations that should be acknowledged. One primary limitation is the relatively small sample size and the relatively short follow-up duration, which was restricted to only 6 months. Further investigations with larger participant cohorts and extended follow-up periods are warranted to establish the long-term effectiveness of BoNT-A in scar reduction. Moreover, considering the potential for optimizing treatment outcomes, exploring the feasibility of repeat injections should be taken into consideration.

Another aspect to consider is the timing of BoNT-A administration. While previous studies have predominantly administered BoNT-A either before wound closure, immediately after wound closure, or during the early postoperative phase [14,19,25,37,38,39], our study uniquely opted for injection 14 days after surgery. This variation in timing could potentially influence the efficacy of BoNT-A, introducing a factor that may have impacted the outcomes observed in our study.

## 4. Conclusions

In conclusion, incoBoNT-A has demonstrated effectiveness in scar prevention. The injections were found to reduce the severity of postoperative scar formation and enhance the overall appearance of scars. To further enhance our understanding and optimize treatment efficacy, future research should explore larger participant cohorts, various injection locations, and different injection timings.

## 5. Materials and Methods

This pilot study was conducted as a prospective, double-blinded, separate-scar side, randomized placebo-controlled trial at Ramathibodi Internaltional Trainging Center for Laser Therapy, Ramathibodi Hospital, Mahidol University, Bangkok, Thailand which involving 15 patients who had undergone female-to-male subcutaneous mastectomy at over a period of approximately 10 months (July 2021 to April 2022).

### 5.1. Study Subjects

This study included female-to-male patients aged 18 years and above. Exclusion criteria encompassed the following: a known history of allergy to BoNT-A, BoNT-A injection within the past 6 months, a history of neurological disease or deficit, concurrent severe underlying or uncontrolled diseases such as malignancies or uncontrolled comorbidities, and the presence of skin infection in the treated area.

### 5.2. Randomization and Blinding

A block randomization method was employed to allocate patients into the following two groups: one receiving incoBoNT-A and the other receiving 0.9% normal saline injections, administered on the right or left surgical scar of the chest, respectively. To ensure blinding, patients and investigators were kept unaware of the treatment assignments. The injections of both incoBoNT-A and 0.9% normal saline were administered by the same independent dermatologist who participated in maintaining the blinding process. The randomization codes were securely sealed and remained undisclosed until the study’s conclusion. This approach ensured that patients, investigators, and research personnel remained blinded throughout the study duration.

### 5.3. Treatment Protocol

In this study, a total of 50 units of Incobotulinumtoxin A (Xeomin, Merz Inc., Frankfurt, Germany) was used, diluted with 1 mL of 0.9% normal saline. The injections were administered 14 days post-surgery, after the removal of stitches and drainage tubes. The injection sites were spaced 1 cm apart along the entire scar, with 5 units per injection point, following a zigzag pattern (based on established best practices) as illustrated in Figure 2. A 30G needle was used for the injections. For the corresponding scar side, 0.9% normal saline in the same quantity was administered using the identical injection pattern.

Throughout the study, patients were allowed to utilize silicone gel, silicone sheets, or pressure garments on the scar. However, they were prohibited from receiving intralesional steroid injections or laser treatments.

### 5.4. Outcome Measurement

The postoperative scar was assessed at the following four different time points: 2 weeks, 1 month, 3 months, and 6 months post-surgery. It was important to schedule a follow-up appointment as early as 2 weeks since the full effects of BoNT-A typically become evident around this time. Also, if any adverse events were to occur, they would likely be detected within the first 2 weeks. The 3- to 6-month follow-up visits were scheduled during the period when postsurgical scarring typically occurs. High-resolution digital photographs (Canon 600D, Tokyo, Japan) were taken under consistent room lighting conditions.

Two independent and blinded dermatologists performed the scar assessments. They evaluated the severity of the scar using the Vancouver Scar Scale (VSS) score, which encompassed parameters including pigmentation, vascularity, pliability, and scar height [40]. Additionally, we used the Patient and Observer Scar Assessment Scale (POSAS), which included ratings from both observers and patients themselves [41]. The mean score from the evaluations of the two dermatologists was employed for subsequent analysis. In order to decrease potential discrepancies of the data assessment, dermatologists who regularly assess scar severity using these scores were requested to evaluate scar severity. Potential side effects were carefully monitored during each visit. Moreover, patient satisfaction was assessed using a 10-cm scale at the end of the study.

For objective assessments, the Colorimeter (DSM-II ColorMeter®, Cortex Technology, Denmark) was utilized to evaluate the scar’s coloration. As a reference value, normal skin adjacent to the scar was measured at the beginning of the study. The measurement process involved these three parameters [42]:

L*: Represents the lightness of the color, where L* = 0 indicates black and L* = 100 indicates white.
a*: Indicates redness and its position between magenta and green. Values on this scale range from a* = −60 (green) to a* = 60 (magenta).
b*: Indicates yellowness and its position between yellow and blue. Values on this scale range from b* = −60 (blue) to b* = 60 (yellow).

To provide an example, the skin regions of the first model exhibited significantly higher a* and b* values compared to the skin regions of the second model. Additionally, the first model had a notably higher L* value. This outcome indicates that the second model possesses a darker skin tone compared to the first model [43].

### 5.5. Statistical Analyses

An intention-to-treat analysis approach was employed for this study. Descriptive statistics included means and standard deviations (SD) for continuous variables and percentages for categorical variables. The primary outcome was compared between the BoNT-A and 0.9% normal saline groups. To analyze the data, a repeated measures approach using a multilevel mixed-effects linear regression was adopted. The results were presented as mean difference along with their corresponding 95% confident intervals (CIs). All statistical analyses were conducted using STATA version 17.0 (StataCorp^®^. 2021. Stata Statistical Software: Release 17. College Station, TX: StataCorp LCC.), The threshold for statistical significance was set as a *p* value less than 0.05 (two-sided).

## Figures and Tables

**Table 1:** Baseline patient characteristics.

Characteristics	n (%)
Age, years, mean (SD)	30.4 (2.0)
Comorbidities	
Yes	1 (6.7)
No	14 (93.3)
History of keloid/hypertrophic scar in other sites of the body	
Yes	1 (6.7)
No	14 (93.3)
Family history of keloid/hypertrophic scar or abnormal wound healing	
Yes	0 (0.0)
No	15 (100.0)
Current medication	
Yes	1 (6.7)
No	14 (93.3)
Alcohol drinking	
Yes	6 (40.0)
No	9 (60.0)
Smoking	
Yes	7 (46.7)
No	8 (53.3)
Concurrent hormonal taking	
Yes	0 (0.0)
No	15 (100.0)
Fitzpatrick’s phototype	
Type 3	6 (40.0)
Type 4	9 (60.0)
Colorimeter on the normal skin area (mean ± SD)	
L*	61.2 (±2.6)
a*	12.6 (±2.5)
b*	10.4 (±2.1)

Abbreviations: SD, standard deviation.

**Table 2:** Baseline assessment scores before intervention for incoBoNT-A and control group.

Assessment Tools (Score Range)	incoBoNT-A Group	Control Group	*p* Value
	n = 15	n = 15	
	Mean (SD)	Mean (SD)	
Vancouver scar scale (0–13)	8.07 (0.96)	8.47 (0.52)	0.170
Patient and observer scar assessment scale (POSAS)			
Observer scar assessment scale (OSAS)			
Summary (0–60)	19.73 (4.89)	20.93 (3.39)	0.440
Overall (0–10)	6.20 (1.01)	6.60 (0.51)	0.180
Patient scar assessment scale (PSAS)			
Summary (0–60)	29.27 (5.34)	31.80 (5.00)	0.191
Overall (0–10)	8.13 (1.92)	8.13 (1.92)	1.000
Colorimeter on surgical scar area			
L* (0–100)	54.07 (6.73)	51.22 (4.94)	0.200
a* (−60–60)	14.18 (0.56)	14.45 (0.68)	0.250
b* (−60–60)	16.82 (6.81)	16.49 (0.70)	0.850

Abbreviations: incoBoNT-A, Incobotulinum neurotoxin A; SD, standard deviation.

**Table 3:** Assessment scores comparison between the incoBoNT-A and control group during the follow-up period.

Assessment Tools	incoBoNT-A	Control	Coef. (95% CI)	*p* Value
	Mean (SE)	Mean (SE)		
Vancouver Scar Scale (VSS)				
2 weeks	6.33 (0.25)	6.87 (0.25)	−0.53 (−1.21, 0.15)	0.124
1 month	5.87 (0.25)	6.40 (0.25)	−0.53 (−1.21, 0.15)	0.124
3 months	6.89 (0.26)	7.36 (0.26)	−0.47 (−1.20, 0.26)	0.208
6 months	7.43 (0.26)	8.82 (0.26)	−1.39 (−2.12, −0.66)	<0.001 *
Patient and Observer Scar Assessment Scale (POSAS)				
Observer Scar Assessment Scale (OSAS)				
Summary score				
2 weeks	13.47 (0.83)	14.13 (0.83)	−0.67 (−2.97, 1.64)	0.571
1 month	12.60 (0.83)	13.40 (0.83)	−0.80 (−3.10, 1.50)	0.496
3 months	11.46 (0.89)	13.77 (0.89)	−2.31 (−4.78, 0.17)	0.068
6 months	19.00 (0.89)	21.15 (0.89)	−2.15 (−4.63, 0.32)	0.088
Overall score				
2 weeks	4.00 (0.21)	4.53 (0.21)	−0.53 (−1.12, 0.05)	0.073
1 month	3.27 (0.21)	3.67 (0.21)	−0.40 (−0.98, 0.18)	0.179
3 months	2.48 (0.23)	3.86 (0.23)	−1.38 (−2.01, −0.75)	<0.001 *
6 months	4.48 (0.23)	5.01 (0.23)	−0.54 (−1.16, 0.09)	0.093
Patient Scar Assessment Scale (PSAS)				
Summary score				
2 weeks	13.67 (1.27)	16.07 (1.27)	−2.40 (−5.92, 1.11)	0.181
1 month	11.27 (1.27)	11.13 (1.27)	0.13 (−3.38, 3.65)	0.941
3 months	12.54 (1.36)	12.54 (1.36)	6.64 × 10^−13^ (−3.78, 3.78)	1.000
6 months	25.31 (1.36)	21.62 (1.36)	3.69 (−0.08, 7.47)	0.055
Overall score				
2 weeks	3.80 (0.34)	3.80 (0.34)	−4.00 × 10^−15^ (0.95, 0.95)	1.000
1 month	2.47 (0.34)	3.13 (0.34)	−0.67 (−1.61, 0.28)	0.168
3 months	1.54 (0.37)	2.46 (0.37)	−0.92 (−1.94, 0.09)	0.076
6 months	4.85 (0.37)	4.38 (0.37)	0.46 (−0.56, 1.48)	0.374
Scar color assessment by Colorimeter				
L* (lightness)				
2 weeks	57.30 (0.94)	55.45 (0.94)	1.85 (−0.74, 4.44)	0.162
1 month	58.68 (0.94)	56.11 (0.94)	2.56 (−0.03, 5.16)	0.052
3 months	58.85 (1.00)	56.44 (1.00)	2.41 (−0.36, 5.18)	0.088
6 months	56.73 (1.00)	55.64 (1.00)	1.09 (−1.68, 3.86)	0.441
a* (redness)				
2 weeks	16.33 (0.48)	15.34 (0.48)	0.99 (−0.33, 2.31)	0.143
1 month	16.31 (0.48)	15.49 (0.48)	0.82 (−0.50, 2.14)	0.224
3 months	15.21 (0.50)	17.18 (0.50)	−1.97 (0.59, 3.34)	0.005 *
6 months	13.36 (0.50)	15.70 (0.50)	−2.33 (−3.71, −0.96)	0.001 *
b* (yellowness)				
2 weeks	15.31 (0.90)	17.54 (0.90)	−2.22 (−4.71, 0.26)	0.079
1 month	11.98 (0.90)	11.47 (0.90)	0.51 (−1.97, 2.99)	0.686
3 months	9.52 (0.96)	12.42 (0.96)	−2.90 (−5.55, −0.25)	0.032 *
6 months	14.93 (0.96)	15.20 (0.96)	0.27 (−2.38, 2.92)	0.843

Abbreviations: incoBoNT-A, Incobotulinum neurotoxin A; Coef., coefficient; CI, confidence interval; SE, standard error; * *p* value < 0.05.

## Data Availability

Not applicable.
